# Anterior Segment Biometry with Phenylephrine and Tropicamide during Accommodation Imaged with Ultralong Scan Depth Optical Coherence Tomography

**DOI:** 10.1155/2019/6827215

**Published:** 2019-02-25

**Authors:** Junna Zhang, Yang Ni, Peng Li, Wen Sun, Mengyun Liu, Dongyu Guo, Chixin Du

**Affiliations:** ^1^Department of Ophthalmology, First Affiliated Hospital, College of Medicine, Zhejiang University, Hangzhou 310003, China; ^2^State Key Lab of Modern Optical Instrumentation, Department of Optical Engineering, Zhejiang University, Hangzhou, Zhejiang 310027, China

## Abstract

**Purpose:**

To investigate the influence of phenylephrine and tropicamide on anterior segment biometry with ultralong scan depth optical coherence tomography (UL-OCT) during accommodation.

**Methods:**

In this study, 20 left eyes of healthy volunteers with a mean ± standard deviation age of 31.05 ± 5.84 years and a mean refraction of −1.16 ± 1.11 diopters (range 0∼−3.0 D) were imaged using UL-OCT after instillation of artificial tears, phenylephrine, and tropicamide in three follow-up trials, respectively. At each follow-up trial, two repeated measurements were performed at states of relax and 5D accommodative stimulation. The dimensional parameters included central corneal thickness (CCT), anterior chamber depth (ACD), pupil diameter (PD), lens thickness (LT), and horizontal radii of the lens anterior and posterior surface curvatures (LAC and LPC).

**Results:**

Tropicamide led to larger pupil, deeper ACD, thinner LT, and flatter crystalline lens surface (*P* < 0.05). Phenylephrine induced an increase in PD (*P* < 0.05), while no significant changes were seen in ACD, LT, LAC, and LPC (*P* > 0.05). CCT did not change after both phenylephrine and tropicamide instillation in this study (*P* > 0.05). Tropicamide induced the loss of accommodation and phenylephrine achieved pupil dilation without affecting the accommodation. PD, ACD decreased, LT increased significantly and the anterior and posterior surface of the lens in a 6.294 mm of diameter optical zone became steeper during accommodation after administration of phenylephrine (*P* < 0.05).

**Conclusion:**

The anterior segment physiology changed after tropicamide instillation. Besides, tropicamide induced the loss of accommodation and phenylephrine preserved the accommodation with a larger pupil. And, the anterior and posterior surface of lens in a 6.294 mm of diameter optical zone became steeper during the accommodation.

## 1. Introduction

Pupillary dilation is an important practice for the purpose of tests and treatments in ophthalmology. Clinically, the sympathetic agonist phenylephrine and the parasympathetic antagonist tropicamide are frequently used to achieve it [1, 2]. And it has been observed the anterior segment physiology may change after these mydriatic drops [3–5]. These parameters (such as CCT, ACD) are important for cataract and aphakia refractive surgery evaluation which concern of accommodation and near vision.

The process of accommodation is an extremely complex and delicate physiological mechanism, related to myopia, presbyopia, and other important eye diseases, which is a research hot spot in the field of ophthalmology for nearly a century. It generally considered that human accommodation is released through the change in crystalline lens shape, which is the result of the cooperation of ciliary and zonular [6, 7] and controlled primarily by parasympathetic input [8]. Nevertheless, an evidence shows that there exists a sympathetic innervation of ciliary muscle [9]. Multiple studies have been performed to analyze the effect of phenylephrine and cycloplegia on the accommodation [10–12]. But, their result was not coincident. Optical coherence tomography provides a high resolution and noninvasive method to image the eye in vivo [13]. And, the UL-OCT can observe the whole anterior segment including the cornea, iris, anterior chamber, and crystalline lens. Currently, UL-OCT has widespread use to study the structural changes of the anterior segment during accommodation [14–16].

In this study, we used UL-OCT to investigate the influence of phenylephrine and tropicamide instillation on anterior segment biometry. Besides, we also documented the parameter changes during accommodation after the two drugs.

## 2. Materials and Methods

All subjects were recruited from the First Affiliated Hospital of Zhejiang University from October 2016 to June 2017. All participants had a best corrected visual acuity of 20/20 or better. Exclusion criteria were history of laser treatment, ocular trauma, diseases or any previous ophthalmic surgery or systemic disease, or medication which might affect accommodation or pupil constriction. The volunteers not able to accommodate 5 diopters were also excluded from the study. Twenty left eyes of healthy volunteers (9 men and 11 women) with a mean ± standard deviation age of 31.05 ± 5.84 years and a mean refraction of −1.16 ± 1.11 diopters (range, 0∼−3 D) were enrolled in this study. Each subject was provided with significance, experimental methods, and possible risks about the study. Informed consent was obtained from each subject, and they were all treated in accordance with the tenets of the Declaration of Helsinki. This study was approved by the Ethics committee of the first affiliated hospital, college of medicine, Zhejiang university.

Their left eyes were tested after instillation of artificial tears (0.5% Carboxymethylcellulose Sodium Eye Drops, Allergan), 1.0% phenylephrine, and 0.5% tropicamide (Tropicamide Eye Drops, Bausch & Lomb) with one drop at 5-minute intervals for 3 times during the three visits, respectively, recorded as Groups I–III. And, each visit at an interval of at least 3 days was scheduled for each subject.

The UL-OCT system and custom algorithms were described in the previous studies, and the good repeatability and reliability of the system have been confirmed [14]. All OCT images were obtained in a dim examination room; each subject sat in front of the slit-lamp and looked forward at the internal fixation target “E” using the left eye, the right eye was covered with a patch. Negative or positive lenses were placed in the lens holder to compensate for the subject's spherical ametropia for near-equivalent spherical refractive correction to achieve relax accommodation. Afterwards, −5.0 D lenses were added to the lens holder to stimulate the physiologic accommodation. Measurements were conducted after 30 minutes with the third drop of phenylephrine or tropicamide which allowed larger pupils. During each visit, the OCT images of the eye were measured while relax and −5.0D stimulating accommodation. While the specular reflection of the corneal apex at the horizontal and vertical meridians being visualized, left and right chamber angles were aligned at the horizontal meridian; meanwhile, the anterior segment of the eye could be seen clearly, and the image was acquired. At least two repeated measurements were performed in each state, and the mean of the 2 results was taken for further statistical analysis. All images were collected by the same operator.

The dimensional parameters in this study included corneal thickness (CCT), anterior chamber depth (ACD), pupil diameter (PD), lens thickness (LT), and horizontal radii of the lens anterior and posterior surface curvatures (LAC and LPC), which were measured on the basis of the related studies [14, 16–18]. The boundaries of the cornea and lens were outlined semimanually to achieve the anterior segment biometry. The position of the anterior and posterior surface of the lens on horizontal meridian within 6.294 mm was traced; additionally, the radius of crystalline lens was determined by measurements that permitted circular fitting to the anterior and posterior lens surface. The optical distortion correction algorithm was based on Snell's principle, and the refractive indices for the cornea was taken as 1.376, the aqueous humor as 1.336, and the crystalline lens as 1.416. The distal limit of each angle recess was identified on the cross-sectional image with the corneal apex. A perpendicular line was drawn from recess-to-recess and extended from the median point through the cornea. Then, CCT, ACD, and LT were calculated along this perpendicular line. And, PD was measured as pupil margin-to-margin distance.

Statistical Procedures for the Social Sciences (SPSS for Windows 20.0, SPSS Inc., Chicago, IL, USA) was used for all statistical analyses. All data were expressed as mean ± standard deviations. After determining that the data for each variable were normally distributed, paired *t*-tests were used for comparison between the measured parameters of the same accommodative state in different follow-up groups and the 0 D and 5.0 D accommodative states in the same group. *P* values less than 0.05 were considered statistically significant.

## 3. Results

**Figure 1:**
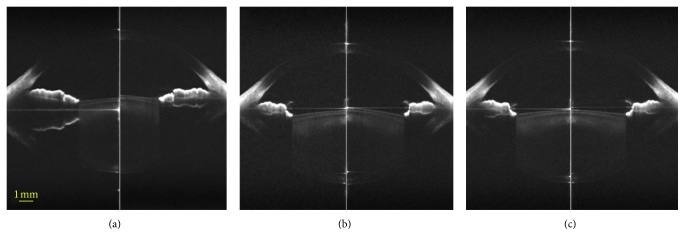
Corrected OCT images at the relax (left) and accommodative states (5.0 D, right) in a 26-year-old female with −1.25 D myopia with (a) artificial tears, (b) phenylephrine, and (c) tropicamide. These three images were corrected only at both corneal surfaces.

The whole anterior segment of the eye was clearly visualized (Figure 1). The lens central position moved forward (anteriorly) during accommodation after instillation of artificial tears and phenylephrine (Figures 1(a) and 1(b)). In contrast, there was no change in the lens central position with tropicamide between the accommodative states (Figure 1(c)). Both phenylephrine and tropicamide led to a larger pupil (Figures 1(b) and1(c)).

Both phenylephrine and tropicamide cause significant mydriasis after instilling 30 minutes in relax status (*P* < 0.001, Table 1). There were no significant changes in ACD, LT, LAC and PLC after administration of phenylephrine (Table 1). And tropicamide led to deeper ACD, thinner LT and flatter crystalline lens surface (*P* < 0.05, Table 1). CCT did not change after both phenylephrine and tropicamide instillation in this study (*P* > 0.05, Table 1).

In Groups I and II, PD, ACD, LAC, and LPC decreased and LT increased significantly during accommodation (*P* < 0.05), while the CCT did not change between accommodative states (paired *t*-test, *P*=0.134, Table 2). In Group III, there was no significant different changes in all parameters after −5.0 D accommodative stimulation (*P* > 0.05, Table 2).

## 4. Discussion

To date, the most widely accepted mechanism of accommodation by ophthalmologists includes two theories: Helmholtz's theory and Schachar's theory. Helmholtz postulated that, during accommodation the ciliary muscle contracts, reducing tension on the zonules and allowing the periphery and center of the lens both become steeper [19, 20]. However, Schachar et al. proposed that when the ciliary muscle contracts during accommodation, the tension on the anterior and posterior zonules reduces and the tension of equatorial zonules increases, making the central lens become steeper and the peripheral lens flatter [21, 22]. So, a certain disagreement of the two accommodative mechanisms is the regulation of the peripheral lens during accommodation. However, most studies cannot analyze the whole lens through the small pupil. In the previous studies, we analyze the lens curvature only of the central 3 mm zone [14, 16]. In our present study, in a 6.294 mm of diameter optical zone after 1.0% phenylephrine, LAC reported in the unaccommodated state (11.475 ± 0.776 mm); LPC (6.190 ± 0.491 mm) are comparable to the well-established reports by Martinez-Enriquez et al. [23] who also measured the lens with one drop of phenylephrine (LAC = 11.65 ± 1.17 mm and LPC = 6.33 ± 0.48 mm) using 3-dimensional OCT and the values reported by Dubbleman and Van der Heijde [24] (LAC = 11.24 mm and LPC = 5.9 mm) using corrected Scheimpflug imaging. We found that the lens curvature radius in a 6.294 mm of diameter optical zone decreased significantly and the lens thickness increased during accommodation, which are in agreement with. Martinez-Enriquez et al. [23] and Hermans et al. [25]. However, the lens surface is aspheric and the value *k* changes with ages and accommodation [26]; the curvature radius obtained by fitting the circular curves may not completely express the anterior and posterior surface state of lens, especially in the periphery. Besides, an error may have been introduced during the correction in the periphery of the lens. So, the change of the real peripheral lens during accommodation cannot be obtained. Therefore, the arguments regarding the accommodative mechanism still have not been clarified.

We demonstrated that phenylephrine induced an increase of 1.917 ± 0.343 mm in pupil size (*P* < 0.05), and there was no significant difference in other parameters. Furthermore, compared to relax status, PD and ACD decreased, LT increased, and LAC and LPC were significantly smaller under 5 D accommodative status (*P* < 0.05). Therefore, our study suggested phenylephrine could induce pupil dilation without affecting the accommodation. This echoes the previous experiments [11, 12, 27, 28], which showed phenylephrine has no effect on the accommodative system performed by objective measurement methods. So, the result is very useful to calculate the phakia IOL and prospect potential risk of glaucoma or cataract after surgery in high myopia. The accommodative or superior IOL evaluation will be more accurate according these parameters under accommodation preservation. Also, phenylephrine can be used to achieve mydriasis in the ocular fundus examination, with which the subjects are still able to work and study.

Our results showed that 0.5% tropicamide got a larger pupil than 1% phenylephrine, an increase of 2.231 ± 0.544 mm. However, the previous studies showed different doses of tropicamide and phenylephrine had different pupillary dilation effects [10, 29]. And, tropicamide led to deeper ACD, thinner lens thickness, and flatter crystalline lens surface compared to Group I in the relaxed state. We suggest that the eyes failed to be relaxed completely in Group I due to the tonic accommodation and the proximal accommodation. We illustrated an increase in ACD after cycloplegia that is compatible with the previous studies [3–5]. Attention should be given in the change of the parameters after cycloplegia in accommodating IOLs implantation and other intraocular refractive surgery evaluation. Ciliary muscles are paralyzed after tropicamide instillation, which company with the complaint of blurring of the fixation stimulus “E.” And, all parameters cannot be changed, even if given a stimulus of 5D. In our study, there was no significant difference in parameters during the relaxed and accommodative states in Group III (*P* > 0.05). So, the ciliary muscle is the initial point and the lens is the target spot for accommodation.

We found no difference in CCT after phenylephrine and tropicamide application (*P* > 0.05), which is compatible with Palamar et al. [5]. However, Chang et al. [4] and Gao et al. [30] demonstrated increment of CCT after tropicamide instillation. We suspect that the result in our measurements could be attributed to the systematic measurement error; therefore, a larger sample size is needed.

Although we applied real-time views of vertical and horizontal scans on image alignment, the lack of proper fiducial markers for positioning the eye may still induce an image registration error. In the future study, the image processing algorithms including automatic segmentation, registration, and distortion correction need to be improved to construct a parametric model to obtain the lens peripheral region through a pupil larger than 6 mm. Meanwhile, the further studies that observe the ciliary muscle and crystalline lens simultaneously will help gain a better understanding of the mechanism of the accommodation.

## 5. Conclusion

In conclusion, the UL-OCT system successfully measured the dimensional changes of the anterior segment with phenylephrine and tropicamide during accommodation. And, tropicamide instillation made alterations in the anterior segment parameter and induced the loss of accommodation, while phenylephrine preserved accommodation with the pupil dilation. We also documented the radius of the lens surface curvatures in a 6.294 mm pupil size became smaller during accommodation and the understanding of accommodative mechanism will be helpful in treating presbyopia, designing the accommodating IOLs, and controlling myopia progress.

## Figures and Tables

**Table 1:** Parameters in the relax state of Groups I–III.

Group	CCT (mm)	ACD (mm)	LT (mm)	PD (mm)	LAC (mm)	LPC (mm)
I	0.547 ± 0.033	2.862 ± 0.267	3.872 ± 0.254	5.596 ± 0.358	11.384 ± 0.622	6.165 ± 0.372
II	0.552 ± 0.029	2.861 ± 0.252	3.873 ± 0.236	7.513 ± 0.325	11.475 ± 0.776	6.190 ± 0.491
*P*	0.105	0.985	0.972	0.000	0.563	0.830

I	0.547 ± 0.033	2.862 ± 0.267	3.872 ± 0.254	5.596 ± 0.358	11.384 ± 0.622	6.165 ± 0.372
III	0.551 ± 0.028	2.980 ± 0.248	3.740 ± 0.242	7.827 ± 0.417	11.993 ± 0.635	6.483 ± 0.314
*P*	0.140	0.000	0.001	0.000	0.008	0.000

**Table 2:** Parameters in the relax (Re.) and accommodative states (Acc.) of Groups I–III.

Group	State	CCT (mm)	ACD (mm)	LT (mm)	PD (mm)	LAC (mm)	LPC (mm)
I	Re.	0.547 ± 0.033	2.862 ± 0.267	3.872 ± 0.254	5.596 ± 0.358	11.384 ± 0.622	6.165 ± 0.372
	Acc.	0.547 ± 0.033	2.713 ± 0.234	4.056 ± 0.255	4.866 ± 0.362	9.311 ± 0.409	5.419 ± 0.365
	*P*	0.203	0.000	0.000	0.000	0.000	0.000

II	Re.	0.552 ± 0.029	2.861 ± 0.252	3.873 ± 0.236	7.513 ± 0.325	11.475 ± 0.776	6.190 ± 0.491
	Acc.	0.554 ± 0.030	2.712 ± 0.234	4.052 ± 0.225	7.220 ± 0.404	9.681 ± 0.901	5.626 ± 0.367
	*P*	0.134	0.000	0.000	0.000	0.000	0.000

III	Re.	0.551 ± 0.028	2.980 ± 0.248	3.740 ± 0.242	7.827 ± 0.417	11.993 ± 0.635	6.483 ± 0.314
	Acc.	0.552 ± 0.029	2.963 ± 0.234	3.755 ± 0.295	7.830 ± 0.490	11.909 ± 0.282	6.411 ± 0.329
	*P*	0.368	0.467	0.514	0.891	0.445	0.127

## Data Availability

The measurement data used to support the findings of this study are available from the corresponding author upon request.
